# Food Neophobia, Odor and Taste Sensitivity, and Overall Flavor Perception in Food

**DOI:** 10.3390/foods10123122

**Published:** 2021-12-16

**Authors:** Sharon Puleo, Ada Braghieri, Corrado Pacelli, Alessandra Bendini, Tullia Gallina Toschi, Luisa Torri, Maria Piochi, Rossella Di Monaco

**Affiliations:** 1 Department of Agricultural Sciences, Food Science and Technology Division, University of Naples Federico II, 80055 Portici, Italy; sharon.puleo@unina.it (S.P.); rossella.dimonaco@unina.it (R.D.M.); 2 School of Agricultural, Forestry, Food and Environmental Sciences, University of Basilicata, 85100 Potenza, Italy; corrado.pacelli@unibas.it; 3 Department of Agricultural and Food Sciences (DiSTAL), University of Bologna, Piazza Goidanich 60, 47521 Cesena, Italy; alessandra.bendini@unibo.it (A.B.); tullia.gallinatoschi@unibo.it (T.G.T.); 4 Sensory and Consumer Science, University of Gastronomic Sciences, 12042 Pollenzo, Italy; l.torri@unisg.it (L.T.); m.piochi@unisg.it (M.P.)

**Keywords:** odor sensitivity, psychological traits, food liking

## Abstract

Smell, which allows us to gather information about the hedonic value of an odor, is affected by many factors. This study aimed to assess the relationship among individual factors, odor sensitivity, and enjoyment, and to evaluate how overall flavor perception and liking in actual food samples are affected by odor sensitivity. A total of 749 subjects, from four different Italian regions, participated in the study. The olfactory capabilities test on four odors (anise, banana, mint, and pine), as well as PROP (6-n-prpyl-2-thiouracil) status and food neophobia were assessed. The subjects were clustered into three groups of odor sensitivity, based on the perceived intensity of anise. The liking and intensity of the overall flavor were evaluated for four chocolate puddings with increasing sweetness (C1, C2, C3, and C4). The individual variables significantly affected the perceived intensity and liking of the odors. Even if all of the odor sensitivity groups perceived the more intensely flavored samples as the C1 and C4 chocolate puddings, the high-sensitivity group scored the global flavor of all of the samples as more intense than the low-sensitivity group. The low-sensitive subjects evaluated the liking of the sweeter samples with higher scores than the moderate-sensitive subjects, whereas the high-sensitive subjects gave intermediate scores. In conclusion, odor sensitivity plays a pivotal role in the perception and liking of real food products; this has to be taken into account in the formulation of new products, suitable for particular categories with reduced olfactory abilities.

## 1. Introduction

Smell is one of the primordial senses, useful for the survival and evolution of many animal species, including humans. In fact, it allows us to gather information about the surrounding environment, invokes emotional states and moods, to socialize, to cope with any risks and stress [1], and to avoid potential food hazards [2]. According to Croy et al. [3], high olfactory sensitivity may enable the perception of potential pathogenic risks and contributes to the evolutionary function of disgust as a disease avoidance mechanism [4].

Odor perception consists of a unique dual-sensory process [5]. Orthonasally perceived odors are useful to locate the source of potential food from a far distance [6], to determine its suitability [7,8], and to affect appetite and satiety [9]. Retronasally perceived odors, in conjunction with taste and oral somatosensation, may screen for potential deviation from the concept of a safe known flavor (e.g., fresh milk), thus playing a significant role in the sensory evaluation of food and eating behavior [10], and shaping appetite and food choices [9,11,12,13].

For all of these implications, a low-odor-sensitivity sense of smell has been associated with a higher risk of diet-related diseases, such as obesity [14] and diabetes [15]. In general, the main information conveyed by olfaction is the hedonic value of an odor [16], even when people are not able to recognize or name a smell. The olfactory function, in turn, can be influenced by various factors, such as learning and memory [17], gender, age, injury, disease, physiological state, food neophobia, and body mass index [18,19,20].

The loss in olfactory sensitivity with aging has been stated in many clinical reports, experimental and epidemiological studies [21,22,23] and has been ascribed to structural changes in the aging nose and olfactory system [23]. The decline in olfaction has detrimental effects on multisensory flavor perception, including loss of taste, producing changes in dietary behavior [24], known as “the anorexia of aging,” leading to malnutrition and immune dysfunction [25], while in other circumstances decline in olfaction may also produce an increased intake of sweet and fatty foods [26], leading to elevated risk of chronic diseases.

Additionally, hormones, such as ghrelin, leptin, insulin, and endocannabinoids, affect odor perception and sensitivity, and they have been shown to be affected by the olfactory system [27]. In particular, insulin in the olfactory bulb affects smelling capacity, adiposity, and insulin resistance [28,29] with a strong negative mediating effect of insulin resistance on olfactory sensitivity to food odors [30]. In addition, odor detection is affected by physiological state in terms of body state (e.g., hunger state, and satiety state); there is a sensory-specific decrease in the pleasantness of an odor of an eaten food to satiety, while it is pleasant during hunger [16]. This is probably due to the increase in olfactory perception during hunger states and the decrease during states of satiety [28]. Other findings have reported that olfaction may be desensitized in individuals with obesity [31,32]. Indeed, many studies related high BMI with olfactory dysfunction [20,31], suggesting a possible link between a poorer sense of smell and overeating. On the other hand, Stafford and Welbeck found that consumers with a high BMI were better at detecting food odors. Also, individuals with obesity rated food odors as more pleasant than people of normal weight [33].

As for gender effect, although there are conflicting findings [34], most studies suggest that women have a greater olfaction performance than men in terms of odor detection, identification, discrimination, and memory [34,35]. Many mechanisms involved in the gender differences in olfaction have been assumed, although the most established theory is related to the close relationship between reproductive hormones (particularly estrogen) and human olfactory function. However, other studies have suggested that odor detection ability [36] and odor identification [37] are similar between the genders.

Differences in odor sensitivity have been related to food neophobia, in particular, food-neophobic subjects smelled odor samples less vigorously than food neophilics [38]. These findings may be considered as an attempt made by neophobics to avoid any potential odor-related experience with foods [39].

Furthermore, Demattè et al. [18] showed that neophobics were less able in odor identification than neophilics. However, Menghi et al. [40] concluded that the conflicting relationship between neophobics and new food may be led by higher levels of arousal toward foods, rather than different chemosensory functions.

Although few studies have assessed the relationship between odor and taste sensitivity [41,42], most of them agree that olfaction plays a fundamental role in the tasting of food [43] and, in agreement with Doty and Kamath [23], it is the most important contributor to the flavor of foods and beverages [44].

According to Skrandies and Zschieschang [42], thresholds for odors and sweet or salty taste are correlated; Piochi et el. [45] confirmed this statement, finding a lower but significant correlation between systems with a different peripherical anatomical localization (oral vs. nasal, e.g., taste index vs. odor intensity index). According to these authors, PROP and “supertasting” should be extended beyond the super-tasting of PROP bitterness to retronasal olfaction [46], as an olfactory role has been shown in the modulation of PROP and oleic-acid sensitivity [47], and the non-tasters were less sensitive to diacetyl than the tasters and super-tasters were [48].

The differences in responsiveness to oral sensations and nasal chemesthetic activity may also affect liking [49,50] as more sensitive subjects perceive smaller variations in food product composition more easily, and, consequentially, have greater changes in liking, compared to low-sensory-responsive subjects [45]. Ramaekers and colleagues [12] found a relationship between odor perception and appetite. However, little is known about the relationship between odor sensitivity, flavor perception and liking in real food products.

Based on these considerations and premises, the present study has a dual purpose, as follows: (a) To assess the relationships among individual factors (such as gender, age, food neophobia, and provenance) and odor sensitivity and liking; (b) To evaluate how odor sensitivity affects consumer perception of overall flavor and liking in actual food samples.

## 2. Materials and Methods

### 2.1. Research Overview

This study is part of the broader *Italian Taste* project, aimed at investigating influences on food choice and preferences in a large population sample [51]. That multisession study consisted of a questionnaire session at home and one-on-one testing in the sensory laboratories over two days. A selection of data, collected in four research units involved in the project, are presented here.

The study was conducted in agreement with the guidelines of the Declaration of Helsinki and the Italian ethical requirements on research activities and personal data protection (D.L. 30.6.03 n. 196). The study protocol was approved by the ethics committee of Trieste University (prot. n. 25138; 9 June 2015) where the genetic unit of the Italian Taste project is based. Informed consent was obtained from all subjects involved in the study.

### 2.2. Subjects

**Figure 1:**
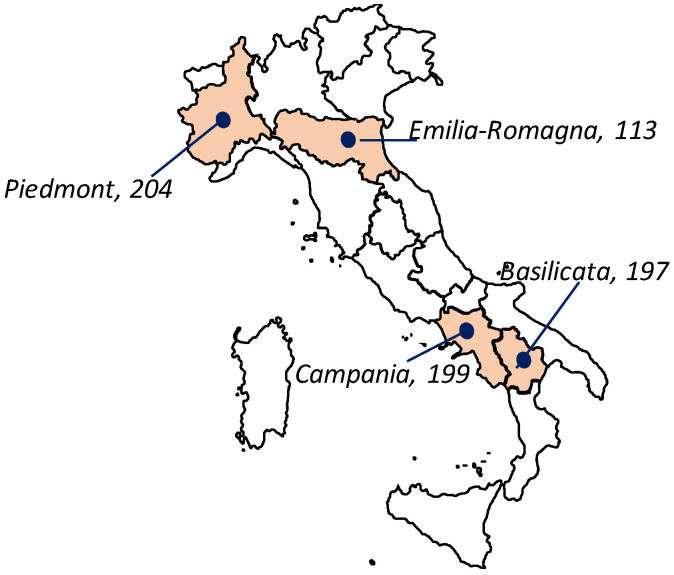
Provenance of subjects involved in the study.

A total of 749 subjects (18–65 years, 420 females and 329 males), from four different Italian regions (197 from Basilicata, 199 from Campania, 113 from Emilia-Romagna, and 204 from Piedmont) participated in the study (Figure 1). Subjects were divided into three age groups (18–30; 31–45; 46–65 years). Data on the socio-demographic information and smoking habits (non-smoker; ex-smoker; current smoker) were collected. Subjects were also asked to declare how they judge their sense of smell (lower than normal/normal/higher than normal) and to state their weight (in kg) and height (in cm), from which their body mass index (WHO/Europe) was calculated.

### 2.3. Test of Olfactory Capabilities

Four pure odorants (anise, banana, mint, and pine) stimulating nasal chemesthesis were tested, as follows: l-menthol stimulating the TRPM8 transient receptor potential ion channels [52], trans-anethole stimulating the TRPA1 [53], amyl acetate stimulating the TRPA1, and (+)-α-terpineol stimulating the TRPA1 [53]. The odors were selected from those included in the European test of olfactory capabilities (ETOC) [54] and presented using cardstocks designed for the project ‘‘The Prevalence of Olfactory Disorders in France” (Project DEFISENS–PREVAL–OLF). The criteria used to select the odors were that they had to be described as an irritant and contain compounds known to activate chemesthetic receptors (in particular, TRPV1 and TRPA1) and they had to differ from one another for the assumed degree of familiarity. Odorant molecules were trapped in tight microcapsules (aminoplast type, diameter: 4–8 micro). The microcapsule-based ink was printed on cardstock (SILK-250 g; dimension: 11 cm × 21 cm). Each odorant was printed on a delimited area (2 cm diameter disc). The release of the odor was done simply by rubbing the printed microcapsule reserve. Participants were asked to smell the cardstock containing the odorant and identify the detected odor by a multiple-choice test (four options); in addition, they had to rate their odor liking (1 = extremely disliked; 9 = extremely liked), the perceived odor intensity (1 = extremely weak, 9 = extremely strong) and irritation (1 = not at all irritant, 9 = extremely irritant). The odorants were presented in a randomized order and a 1 min break was observed between odorants.

### 2.4. PROP Status

PROP taster status was assessed using a 3.2 mm PROP solution, prepared by dissolving 0.545 g/L of 6-n-propyl-2-thiouracil (Sigma-Aldrich, Burlington, MA, USA) into deionized water. Subjects rated the intensity of bitterness of two identical samples by using the generalized labelled magnitude scale (gLMS) [51]. Sample presentation and evaluation procedure were reported in Bartoshuk et al. [55]. PROP taster status of each subject was defined based on the average rating of the two replicates. Subjects were classified for responsiveness to PROP bitterness into three classes, applying previously used cut-off values [45], as follows: no-taster = NT (ratings on gLMS ≤ moderate, 17), medium-taster = MT (17 < ratings on gLMS < 53), and super-taster = ST (ratings on gLMS ≥ very strong, 53).

### 2.5. Liking and Overall Flavor Intensity in Real Food Products

A dark chocolate pudding was prepared by dissolving a pudding mix in water, without sugar (Cameo S.p.A., Desenzano del Garda, Brescia, Italy), with the added bitter cocoa powder (Perugina, Nestlè, Italy) which was selected for the study. Four samples of the product were prepared by adding different amounts of sucrose (C1 = 38 g/kg; C2 = 83 g/kg; C3 = 119 g/kg; C4 = 233 g/kg) to the base. The addition of sucrose was expected to increase sweetness, decreasing bitterness and astringency, and modifying the overall flavor perception.

The liking and intensity of the overall flavor were evaluated on two separate days. During the first session, participants were asked to rate their liking for each sample by using the labeled affective magnitude scale, LAM [56]. During the second session, participants evaluated the intensity of the overall flavor for each of the samples using the gLMS [55]. Instructions for the use of both scales prior to tasting were provided. In each session, the samples (15 g each) were served at room temperature and presented simultaneously in plastic cups coded with 3-digit numbers. The four levels of target stimulus were tested in random order. The participants were instructed to eat the entire amount provided prior to rating liking/intensity. An interval of 90 s was imposed between tastings, during which water was provided for palate cleansing.

### 2.6. Food Neophobia Assessment

Food neophobia was assessed using the food neophobia scale (FNS) developed by Pliner and Hobden [57]. The FNS consists of ten statements (regarding the individual’s willingness to try new foods) evaluated with a 7-point agreement scale (1 = I strongly disagree; 7 = I strongly agree). The individual FNS scores were computed as the sum of ratings given to the 10 statements, after the neophilic items had been reversed; thus, the scores theoretically ranged from 10 to 70, with higher scores reflecting higher FN levels. The FNS quartile distribution was calculated, and respondents were divided into the following 3 groups according to their FN level [58]: low (FN score ≤ 19), moderate (19 < FN score < 36) and high (FN score ≥ 36).

### 2.7. Data Analysis

**Figure 2:**
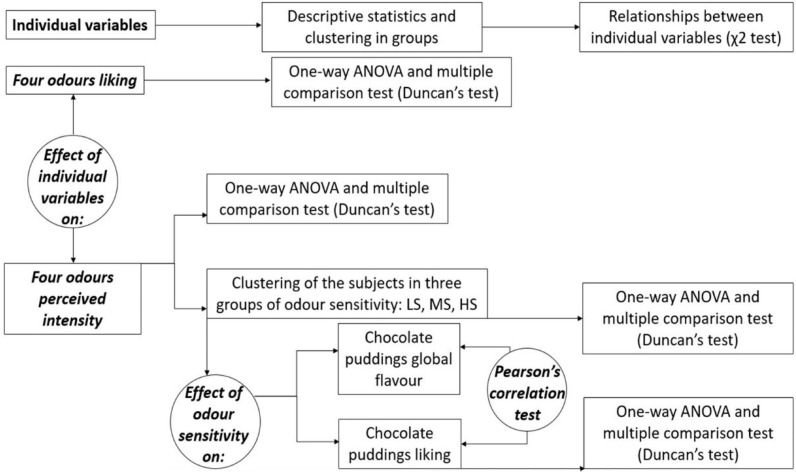
Scheme of data analysis.

The data analysis approach is described in Figure 2 and also detailed below.

Individual variables related to gender, provenance, age, BMI, smoking habits, and self-reported smell were elaborated through descriptive statistics. The approaches used to treat the food neophobia data and the PROP status is explained in the previous paragraphs. To measure the odor sensitivity, considering that the anise odor collected fewer correct answers than the other three odorants, the recognition of anise was used as a factor for the subjects’ clustering into groups of odor sensitivity. Therefore, subjects were clustered in three groups of odor sensitivity, based on the perceived intensity of anise odor; subjects who did not recognize the odor were clustered in the low-odor-sensitivity group (LS). Then, using the median of the intensity scores as cut-off, subjects were clustered in the moderate-odor-sensitivity group (subjects with scores lower than the median value) and the high-odor-sensitivity group (subjects with a score higher than the median value). Relationships among the individual variables were explored by running chi-square tests. The differences between the four odors in terms of liking and perceived intensity were analyzed by one-way ANOVA and a multiple comparison test (Duncan’s test). The effect of individual variables on perceived intensity and liking of the four odors was tested by means of one-way ANOVA and a multiple comparison test (Duncan’s test). Repeated measures ANOVA and a multiple comparison test (Duncan’s test) were used to evaluate whether differences among the four chocolate puddings (used as a repeated factor) were statistically significant in terms of global flavor perception and liking. Pearson’s correlation was used to investigate the relationship between global flavor perception and liking scores of the four chocolate puddings. Finally, the three groups of odor sensitivity were used as fixed variables in repeated measures ANOVA models to explore their influence on global flavor perception and liking of the four chocolate puddings. The XLSTAT statistical software package version 2016.02 (Addinsoft) was used for data analysis. Significance was set for *p* values < 0.05.

## 3. Results

### 3.1. Subjects Description

The main characteristics of the 749 subjects are described in Table 1.

### 3.2. Relationships among Individual Variables

The relationships among all explored individual variables were studied by running a chi-square test (Table 2).

As it can be observed in Table 2, the BMI was significantly related to gender and age.

In particular, the underweight group was more represented by the females (90%) than the males (10%); the normal-weight group was more represented by the females (64%) than the males (36%); the overweight group was more represented by the males (64%) than the females (36%), while males and females were equally distributed in the group with obesity. Considering the relationships between BMI and age, instead, the underweight group was more represented by the young-adult subjects (55%) than the adult (28%) and old-adult (17%) subjects; similarly, the normal-weight group was more represented by the young-adult subjects (39%) than the adult (33%) and old-adult (27%) subjects; the overweight group was more represented by the old-adult subjects (37%) than the young-adult (30%) and adult (33%) subjects; similarly, the group with obesity was more represented by the old-adult (42%) than the young-adult (21%) and adult (37%) subjects.

The smoking habits were significantly related to age. In particular, the never-tried group was more represented by the young-adult subjects (39%) than the adult (35%) and old-adult (27%) subjects; the not-smoking group was more represented by the old-adult subjects (54%) than the young-adult (14%) and adult (33%) subjects; the regularly-smoking group was more represented by the young-adult subjects (46%) than the adult (31%) and old-adult (23%) subjects.

The self-reported smell was significantly related to BMI. In particular, the less-than-normal-smell group was more represented by the normal-weight subjects (73%) than the underweight (0%), the overweight (27%) and the individuals with obesity (0%); similarly, the normal-smell group was more represented by the normal-weight subjects (61%) than the underweight (3%), the overweight (27%) and the individuals with obesity (8%); the more-than-normal smell group was more represented by the normal-weight subjects (60%) than the underweight (10%), the overweight (20%) and the individuals with obesity (10%).

The PROP status was significantly related to gender and age. In particular, the super-taster group was more represented by the females (66%) than the males (34%), while males and females were equally distributed in both the no-taster (females 49%, males 51%) and the medium-taster (females 49%, males 51%) groups.

Instead, considering the relationship between PROP status and age, the no-taster group was more represented by the old-adult subjects (37%) than the young-adult (29%) and adult (34%) subjects; the super-taster group was more represented by the young-adult subjects (42%) than the adult (33%) and old-adult (24%) subjects; the three groups of age were equally distributed in the medium-taster group.

Food neophobia was significantly related to gender and age. In particular, the low-neophobia group was more represented by the females (65%) than the males (35%), while males and females were equally distributed in both the medium- and high-neophobia groups.

Considering the relationship between food neophobia and age, the low-neophobia group was more represented by the young-adult subjects (44%) than the adult (33%) and old-adult (23%) subjects; the high-neophobia group was more represented by the old-adult subjects (45%) than the young-adult (27%) and old (28%) subjects; the medium-neophobia group was more represented by the young-adult (36%) and adult (37%) subjects than old-adult subjects (27%).

**Figure 3:**
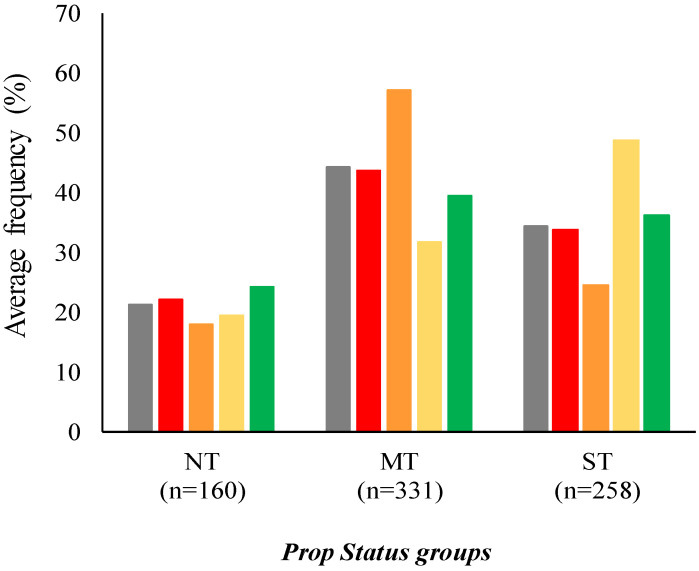
Average frequency (%) of PROP status groups × provenance (■ All subjects (*n* = 749); ■ Basilicata, *n* = 197; ■ Campania, *n* = 199; ■ Emilia-Romagna, *n* = 113; ■ Piedmont, *n* = 240).

Finally, the provenance was significantly related to age, BMI, smoking habits, PROP status and food neophobia. Considering the relationship with age, the group from Emilia- Romagna was more represented by the young-adult subjects (74%) than the adults (9%) and old-adult (17%); the group from Basilicata was more represented by the adults (36%) and old-adult (34%) subjects than the young-adult (30%) subjects; the group from Piedmont was more represented by the adult subjects (39%) than the young-adult (26%) and old-adult (35%) subjects; the three groups of age were equally distributed in the group from Campania. Considering the relationship with the BMI, all of the provenance groups were significantly more represented by the normal-weight subjects (Emilia-Romagna, 79%; Campania, 53%; Basilicata, 55%; Piedmont, 67%) than the other BMI groups. Considering the relationship with the PROP status, the group from Emilia-Romagna was more represented by the super-taster subjects (49%) than the no- (19%) and medium-taster (32%) subjects; the group from Campania was more represented by the medium-taster subjects (57%) than the no- (18%) and super-taster (25%) subjects. The three groups of PROP status were equally distributed in the groups from Basilicata and Piedmont (Figure 3).

**Figure 4:**
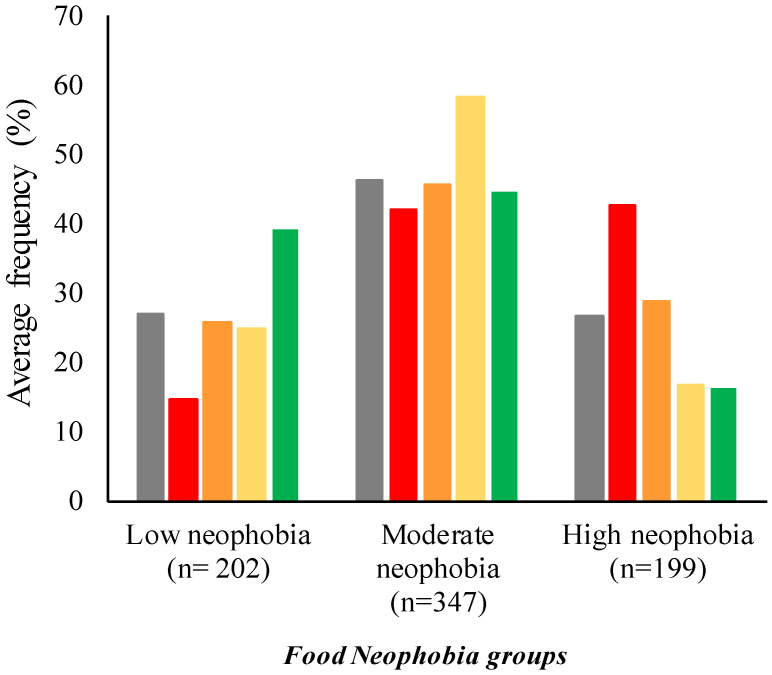
Average frequency (%) of food neophobia groups × provenance (■ All subjects (*n* = 749); ■ Basilicata, *n* = 197; ■ Campania, *n* = 199; ■ Emilia-Romagna, *n* = 113; ■ Piedmont, *n* = 240).

Finally, considering the relationship between the provenance and food neophobia, Figure 4 shows the frequency of the different groups. The more neophobic people were from Basilicata (43%); on the contrary, the more neophilic people were from Piedmont (39%). Finally, people from Emilia-Romagna and Campania were mainly characterized by moderate neophobia (58% and 46%, respectively).

### 3.3. Recognition, Liking, and Intensity Perception of Odors

The olfactory capabilities test was assessed on four odors (anise, banana, mint, and pine). The percentages of correct answers, as well as the liking and perceived intensity scores (mean ± standard deviation) of each odor, are shown in Table 3.

The odor of anise collected fewer correct answers than the other three odorants. For this reason, the recognition of anise was used as a factor for the subjects’ clustering into groups of odor sensitivity, as explained in the data analysis. Moreover, there were no differences among the odors, neither in terms of liking nor in terms of perceived intensity (*p* > 0.05).

**Figure 5:**
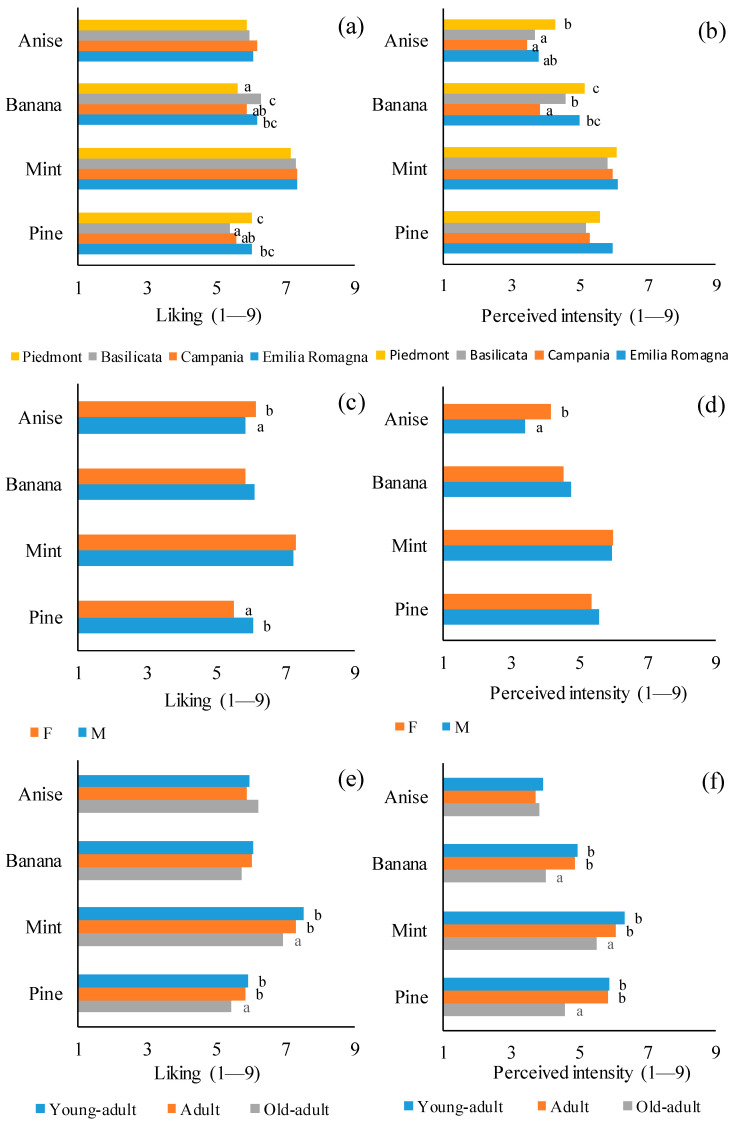

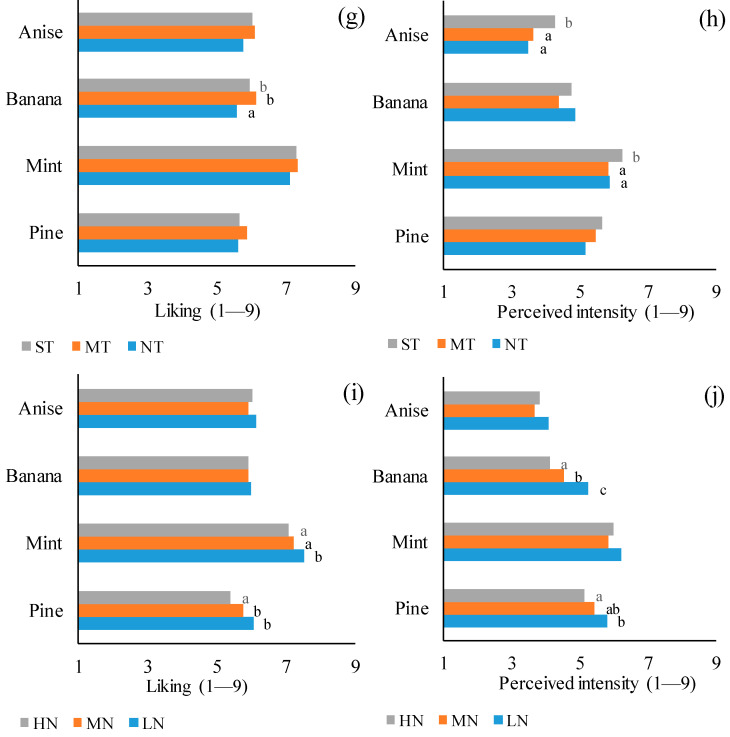
Effect of provenance (**a**,**b**), gender (**c**,**d**), age (**e**,**f**), PROP status (**g**,**h**) and food neophobia (**i**,**j**) on the liking and the perceived intensity. For each graph, at different letters correspond significantly different values (*p* < 0.05).

Each characteristic described in Table 1 was used as an independent variable in one-way ANOVA models to analyze their effect on the liking and the perceived intensity of the four odors. In particular, provenance, gender, age, PROP status, and food neophobia significantly influenced the liking and the perceived intensity (Figure 5).

Therefore, considering the effect of the provenance (Figure 5a), the consumers from Emilia-Romagna and Basilicata liked the banana odor significantly more than the consumers from Piedmont and Campania (*p* = 0.001). On the other hand, the consumers from Piedmont and Emilia-Romagna liked the pine odor significantly more than the consumers from Basilicata and Campania (*p* = 0.002). The banana odor was perceived with higher intensity by the consumers from Piedmont and Emilia-Romagna than the consumers from Campania and Basilicata (*p* = 0.0001). Similarly, the consumers from Piedmont and Emilia-Romagna perceived the anise odor with higher intensity compared to the consumers from Campania and Basilicata (*p* = 0.014, Figure 5b). 

Secondly, gender significantly affected the liking for anise and pine odors. In particular, the females liked the anise odor more than the males (*p* = 0.04), while the males liked the pine odor more than the females (*p* = 0.0001, Figure 5c). Considering the odor perceived intensity, the females perceived the anise odor with a higher intensity than the males (*p* = 0.0001, Figure 5d).

The liking of mint and pine odors were significantly affected by age (Figure 5e). In particular, the liking scores of the young-adult and adult consumers were significantly higher than those given by the old-adult consumers (*p* < 0.05). Also, the perceived intensity scores given by the old-adult consumers were significantly lower than those given by the young-adult and adult consumers for banana, mint, and pine odors (*p* < 0.0001, Figure 5f).

PROP status significantly affected the liking for the banana odor (Figure 5g). In particular, the liking scores of the medium- and super-tasters were significantly higher than those of the no-tasters (*p* = 0.007). Also, the perceived intensity for the anise and mint odors was significantly affected by the PROP status (Figure 5h). In particular, the super-tasters perceived the anise and mint odors with higher intensity compared to the medium- and no-tasters (*p* < 0.05).

Finally, food neophobia significantly affected the liking of the mint and pine odors (Figure 5i). In particular, the mint-liking scores of the low-neophobia consumers were significantly higher than those given by the medium- and high-neophobia consumers (*p* = 0.023), while the pine-liking scores of the low- and medium-neophobia consumers were significantly higher than those given by the high-neophobia consumers (*p* = 0.007). Moreover, the intensity of the banana and pine odors perceived by the low-neophobia consumers was significantly lower than that perceived by the medium- and high-neophobia consumers (*p* < 0.05, Figure 5j).

### 3.4. Effect of Odor Sensitivity on Global Flavor Perception and Liking in Real Food Products

**Figure 6:**
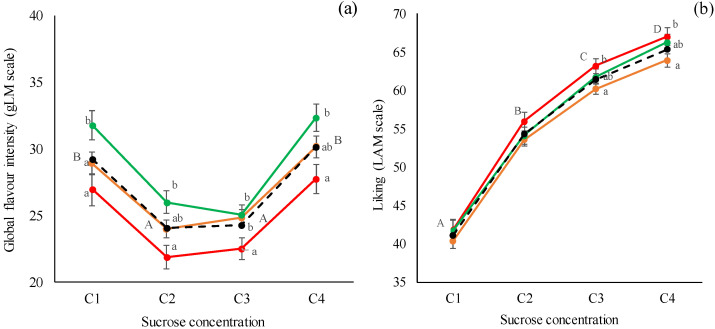
Effect of sucrose concentration (C1 = 38 g/kg; C2 = 83 g/kg; C3 = 119 g/kg; C4 = 233 g/kg) and odor sensitivity (LS, MS, HS) on: (**a**) global flavor intensity and (**b**) liking scores of chocolate pudding. LS = Low-odor-sensitivity (▬); MS = Moderate-odor-sensitivity (▬); HS = High-odor-sensitivity (▬); Average = all the subjects (▬ ▬); Different capital letters indicated significant differences among sucrose concentrations (within groups) (*p* ≤ 0.05); Different small letters indicated significant differences among odor-sensitivity groups (between groups) (*p* ≤ 0.05).

The three groups of odor sensitivity were used as fixed variables in repeated measures ANOVA models to explore their influence on global flavor perception and the liking of the four chocolate puddings (Figure 6a,b).

By considering the global flavor perception (Figure 6a) the same trend was observed for all of the odor sensitivity groups, so the C1 and C4 chocolate puddings were perceived as the more flavored samples if compared with the C2 and C3 chocolate puddings. The odor sensitivity of the participants significantly affected their perceived intensity of global flavor in all of the chocolate puddings. In particular, the HS subjects perceived and scored all of the samples with higher intensity, if compared to LS subjects (*p* < 0.05). The MS subjects gave intermediate intensity scores to the global flavor of chocolate puddings.

When considering the liking scores (Figure 6b), the same trend was observed for all of the odor sensitivity groups as well, in particular for all of the subjects the liking significantly increased when the sucrose concentration increased, and all of the samples, from C1 to C4, were significantly different one to each other.

On the other hand, the odor sensitivity of the participants significantly affected the liking scores of the third and fourth samples. In particular, the LS subjects evaluated the liking of the sweeter samples with higher scores than the MS subjects (*p* = 0.05), whereas the HS subjects gave intermediate scores.

## 4. Discussion

### 4.1. Relationships among Individual Variables

The prevalence of overweightness and obesity is the result of several interacting forces, including age- and sex-specific body mass index (BMI) change. As for BMI and the gender relationship, as expected, a higher percentage of females were underweight and normal weight compared with males, as females are more concerned with healthy diets but are also particularly worried about their body image, thus controlling their weight [59,60,61]. On the contrary, other authors [62] have found that in all age groups, obesity was significantly more frequent in females compared with males.

Although the prevalence of obesity varies remarkably in various populations, sexes, and age groups [63,64], our results agree with other studies, finding that BMI increases with age and reaches its peak at around 55–64 years, then decreases afterwards [62].

The significant relationship between BMI and self-reported olfaction was found in the present study, with the less-than-normal-smell group more represented by normal-weight subjects than underweight-, overweight, and respondents with obesity, as in agreement with Stafford and Welbeck [65]; these authors found that consumers with a high BMI were better at detecting food odors. On the contrary, other studies related high BMI with olfactory dysfunction [20,31], suggesting a possible link between a poorer sense of smell and overeating.

In agreement with Tepper et al. [66], gender and PROP responsiveness were significantly related, and women were more sensitive to PROP bitterness as super-tasters compared to men [67]; this may be due to their higher number of taste buds and fungiform papillae than men [67,68,69,70,71]. However, other reports do not substantiate this difference [72,73,74].

Increasing age was negatively related to PROP bitterness rating. Similarly, Steele et al. [75] found a reduced lingual sensitivity in adults over 60 years of age, compared to those under 40 years. Also, in this case, this reduction may be due to the decreasing of FPD from 9–10 years of age to 50–60 years [71,76,77]. The mechanisms that cause age-associated declines in the peripheral taste structures remain mostly unclear [78]; however, ageing affects fungiform papillae morphology, increasing in diameter with age, and their functioning becomes less vascularized in subjects older than 60 years [76].

The lowered responsiveness to PROP with ageing has been related to the variation of the phenotypic expression of the TAS2R38 gene with age [79]. In addition, Tepper et al. [66] found higher percentages of PROP NT in older subjects (age > 50 years).

However, von Atzingen and Silva [80] did not find any gender or age effect on PROP taster status in Brazilian adults (20–60 years of age).

Although some authors concluded that gender had little or no effect on neophobia [81,82], in other studies the food neophobia scores were lower among women than among men [83], a trend that has also been observed among Swedish adults [84,85] but not among Canadian [57] or Finnish 15-year-old teenagers [86]. This difference between genders may have cultural and social origins, as women are still more often responsible for food preparation compared to men [87] and, thus, they are exposed to foods and various food-related issues more extensively than men, and this diminishes neophobic responses [88].

As to the effect of age, high food neophobia scores were found among the elderly [89]. On the contrary, the lower FNS scores of young people could be attributed to their more exploratory and playful attitude, even towards food, which can promote curiosity towards new foods [90].

### 4.2. Recognition, Liking, and Intensity Perception of Odors

Olfactory perception widely varies among individuals in odor sensitivity [91], hedonic [92,93], semantic processing [94,95,96], and higher olfactory cognition [97,98,99,100]. Odor perception and its hedonic responsiveness are then modulated by stimulus characteristics (e.g., concentration [101,102,103]), previous experience, current physiological status (e.g., prandial state, reproductive status [104]), ageing [105,106], stimulus exposure context (in association with gustatory stimuli [9,107]), and stimulation route (orthonasal vs. retronasal olfaction [108,109,110]). The results of early experience can be seen in the cultural variability of olfactory preferences [111,112] since in different cultures, individuals are exposed to different olfactory experiences during their life.

Indeed, as shown in the results paragraph, provenance significantly affected both the liking and the perceived intensity of some odors. The link between provenance, liking, and perceived intensity could be revealed by familiarity [113] and, therefore, by frequency of use.

Secondly, we also observed significant gender differences in liking and perceived intensity. However, not all of the odors were associated with a greater female/male preference; anise triggered more positive hedonic responses in the females, while pine was liked more by the males. Therefore, gender differences seem to also depend on other factors, such as cultural background and general familiarity. Nevertheless, where gender differences exist, females are usually more sensitive to odors compared to men [114,115]. In our study, indeed, the females perceived the anise odor with higher intensity than the males. Several studies revealed that women care about olfaction more than men do, compared with other sensory modalities [116,117]. Furthermore, women are better at identifying and memorizing odors of various origins, such as food [21,118,119]. This difference may be due to the absolute total number of neuronal and non-neuronal cells, favoring women by 40–50% [120].

Moreover, we also observed a significant effect of age on both the liking and the perceived intensity. Indeed, regarding the liking, it is possible to observe a trend among the three groups of age. In particular, the liking is higher for the young subjects compared to the old subjects. However, how the hedonic responses to odors evolve with age remains unclear today. Indeed, in the study conducted by Wang and colleagues [121] the smells of lavender and spearmint were judged more pleasant by the older subjects, while in the study conducted by Markovic and colleagues [122] there was such an age effect for certain smells (turpentine, garlic, and fish became less unpleasant with age while cloves and rose became more pleasant) but not for others (orange, leather, cinnamon, spearmint, banana, lemon, anise, coffee, apple, pineapple, and licorice). Nevertheless, also in this case, where age differences exist, the young are usually more sensitive to odors than old individuals [22,23,123]. In our study, indeed, the young-adult subjects perceived the banana, mint, and pine odors with higher intensity than the adult and old-adult subjects. Certainly, there are multiple determinants of the olfactory loss in older persons (such as changes in autonomic control of nasal engorgement, increased propensity for nasal disease, cumulative damage to the olfactory epithelium from environmental insults, loss of selectivity of olfactory receptor, etc.), although the relative importance of each is yet to be established [23].

Furthermore, liking and intensity perception of the tested odors were also affected by the PROP status. The no-taster individuals, indeed, liked the banana odor significantly less than the super- and medium-taster individuals. To the best of our knowledge, there are no studies showing the relationships between PROP sensitivity and odor liking, however, considering that banana has a sweet flavor, some speculation can be drawn. Several studies have examined taster status in relation to hedonic ratings for sweet-tasting compounds in model systems. Our results seem to be in contrast with the majority of these studies, which found a link between PROP status and hedonic responsiveness for sweetness, however, the results are conflicting [48,124,125]. On the other hand, the odor perceived intensity scored by the three groups of PROP responsiveness is in line with what was expected. Historically, indeed, it is reported that the ability to taste PROP is associated with higher responsiveness to other stimuli, including those related to the taste [126] and chemical irritants [127]. Our results suggest that this association is true for odor perception as well. Indeed, the super-tasters perceived the anise and mint odors with higher intensity, compared to medium- and no-taster individuals.

Finally, as expected, food neophobia played a significant role in odor liking and perceived intensity. High-neophobic subjects gave lower liking scores to the mint and pine odors and lower perceived intensity scores to the banana and pine odors. These results are in accordance with the studies conducted by Raudenbush and colleagues [38] and Menghi and colleagues [40], which showed that neophobic individuals rated the evaluated odors as less pleasant and perceived them less intensely. A possible explanation of these findings lies in the caution that neophobic subjects put on trying novel olfactory stimuli. Indeed, the odor perceived intensity increases with increases in sniff magnitude [128]. Therefore, a person who is unwilling to try new stimuli probably tends to sniff the odors, paying more attention and, consequently, less vigorously than a person who is willing to.

### 4.3. Effect of Odor Sensitivity on Global Flavor Perception and Liking in a Real Product

In our study, the odor sensitivity of subjects was measured by means of a simplified version of ETOC and anise odorant was used as an index of subject sensitivity. Also, in a study by Lo and colleagues [129], anise was found to be predictive of smell ability. In a study by Limphaibool et al. [130], subjective olfactory tests revealed that 94% of subjects with olfactory dysfunction were not able to identify anise whereas all of the subjects in the control group, without olfactory dysfunction, were able to identify it.

Even if strong evidence has shown that odors are perceived differently when presented in orthonasal versus retronasal ways [131], the results from the current study demonstrated that human odor sensitivity clearly affected the perception of flavor intensity in the chocolate puddings. Despite any differences, the trend was observed among odor sensitivity groups, i.e., all of the groups were able to perceive the differences among the global flavor intensity of the different samples, in the same way, the HS group perceived a higher intensity than the LS group. Thus, our results showed that the sensitivity of orthonasal perception is also positively related to retronasal perception.

In other studies, perception via the orthonasal and retronasal ways are different for the same odorants and mixture of odorants [109,110]. In a study by Delime et al. [132], a sensory panel evaluated the relative contributions of volatiles in a nine-component, commercial strawberry flavor, both orthonasally and retronasally. Compared to the retronasal perception, the orthonasal perception was more sensitive to the removal of all individual volatiles.

In our study, odor sensitivity also affected the liking of chocolate puddings, in particular, the low-sensitive subjects liked the samples with higher sugar concentrations more than the others. No relation was found instead among liking and intensity of global flavor, in fact, in our study, the liking of the chocolate puddings significantly increased with sugar concentration. In a study by Sollai et al. [133], intensity and liking of banana odor mixture was instead positively related. That study also revealed that human perception of single compounds is conditioned by the threshold olfactory performance of the subjects and their abilities to detect single molecular components affect both perceived intensity and liking for the complex aroma.

## 5. Strengths and Limitations

The strength of this study lies with the presentation and exploration of individual variables, including sensory sensitivity, on a large group of consumers (749 subjects) from four different Italian regions. Also, the present study included a real food product, the chocolate pudding, modified with different levels of sugar concentration, which allowed us to investigate the real human perception.

However, our results should be viewed under the limitations of this research. The study, indeed, was somewhat limited by the differences in the gender and age of the subjects within the provenances. Since this limit might especially affect the relationship between gender and age with the PROP and FNS groups, those specific results should not be generalized towards the global population.

## 6. Conclusions

The study showed significant relationships among the explored individual variables. Many of these, in particular provenance, gender, age, PROP status, and food neophobia, significantly affected both the intensity and the liking for the considered odorants.

The anise odorant was the least recognized odor among the consumers involved in the study, thus, it was used for clustering participants into the three different groups of odor sensitivity.

Even if the same trend in perceiving the most flavored chocolate puddings was observed for the three sensitivity groups, the HS subjects always perceived more intense global flavor than the LS subjects.

However, as odor sensitivity affected the intensity of flavor perceived in the chocolate puddings, as well as the liking for them, we can confirm and conclude that smell plays an essential role in affecting flavor perception and liking even in real and complex food products.

This has to be considered in the formulation of new products, suitable for particular categories or age groups with reduced olfactory abilities.

## Figures and Tables

**Table 1:** Characteristics of subjects (*n* = 749) involved in the study.

	Number of Subjects	Females (*n* = 420)	Males (*n* = 329)
Provenance			
*Emilia-Romagna*	113	73 (65%)	40 (35%)
*Piedmont*	240	125 (52%)	115 (48%)
*Campania*	199	110 (55%)	89 (45%)
*Basilicata*	197	112 (57%)	85 (43%)
Age (years)			
*19–30*	269	155 (58%)	114 (42%)
*31–45*	249	134 (54%)	115 (46%)
*46–65*	231	131 (57%)	100 (43%)
Body mass index ^1^			
*Underweight*	31	27 (87%)	4 (13%)
*Normal-weight*	447	284 (63%)	163 (37%)
*Overweight*	209	79 (38%)	130 (62%)
*Obese*	62	29 (47%)	33 (53%)
Smoking habits			
*Non-smoker*	404	234 (58%)	170 (42%)
*Ex-smoker*	137	70 (51%)	68 (49%)
*Current-smoker*	206	115 (56%)	91 (44%)
Self-reported smell			
*<Normal*	27	11 (41%)	16 (59%)
*Normal*	620	348 (56%)	272 (44%)
*>Normal*	102	61 (60%)	41 (40%)
Odor sensitivity			
*Low-sensitivity (LS)*	187	83 (44%)	104 (56%)
*Moderate-sensitivity (MS)*	349	142 (41%)	349 (59%)
*High-sensitivity (HS)*	213	130 (61%)	83 (39%)
PROP status			
*No-taster (NT)*	160	81 (51%)	79 (49%)
*Medium-taster (MT)*	331	168 (51%)	163 (49%)
*Super-taster (ST)*	258	171 (66%)	87 (34%)
FNS			
*Low-Neophobia (LN)*	203	131 (65%)	72 (35%)
*Medium-Neophobia (MN)*	347	173 (50%)	174 (50%)
*High-Neophobia (HN)*	199	116 (58%)	83 (42%)

^1^ Body mass index: Underweight, BMI < 18.5; Normal-weight, 18.5 < BMI < 24.9; Overweight, 25 < BMI < 30; Obese, BMI > 30.

**Table 2:** Relationships among all explored individual variables (*p* values).

Individual Variables	Gender	Age	BMI	SH ^1^	SRS ^2^	PROP	FNS	Provenance
Gender	n.d.							
Age	0.672	n.d.						
BMI	<0.0001	0.005	n.d.					
SH1	0.339	<0.0001	0.422	n.d.				
SRS	0.146	0.436	0.012	0.192	n.d.			
PROP	0.0001	0.025	0.163	0.494	0.577	n.d.		
FNS	0.003	<0.0001	0.068	0.112	0.092	0.430	n.d.	
Provenance	0.162	<0.0001	<0.0001	<0.0001	0.087	0.0001	<0.0001	n.d.

^1^ SH = Smoking habits; ^2^ SRS = Self-reported smell. n.d. = Not detectable.

**Table 3:** Percentages of correct answers, liking and perceived intensity scores (mean ± standard deviation) for four tested odors.

	Anise	Banana	Mint	Pine
Recognition (%)				
*Yes*	75	92	99	88
*No*	25	8	1	12
Liking	6 ± 2	6 ± 2	7 ± 2	6 ± 2
Intensity	4 ± 3	5 ± 2	6 ± 2	5 ± 3

## Data Availability

The data presented in this study are available upon request, from the corresponding author.
